# Anxiety Assessment in Methamphetamine - Sensitized and Withdrawn Rats: Immediate and Delayed Effects

**Published:** 2015-06

**Authors:** Hossein Miladi-gorji, Atefeh Fadaei, Imanollah Bigdeli

**Affiliations:** 1 Laboratory of Animal Addiction Models,Research Center and Department of Physiology, School of Medicine, Semnan University of Medical Sciences, Semnan, Iran; 2 Faculty of Psychology and Educational Sciences, University of Semnan, Semnan, Iran; 3 Faculty of Educational Sciences and Psychology, Ferdowsi University of Mashhad, Mashhad, Iran

**Keywords:** *Anxiety*, *Elevated plus-maze*, *METH dependence*, *METH- sensitized rats*

## Abstract

**Objective:** The anxiety profile in the stimulant-sensitized animals is not clear. Thus, this study was conducted to elucidate the effects of acute and chronic administration of methamphetamine (METH) on the anxiety profile. The aim of this study was to examine whether METH-sensitized rats would show an increase in the expression of anxiogenic-like behaviors and to determine whether a low dose of METH elicits behavioral sensitization.

**Methods:** Rats were repeatedly given METH (2 mg/kg, s.c., once a day for 14 days), and the immediate and delayed effects of METH on the anxiety profile was compared considering 30 minutes (min) and 120 min after injections in METH-sensitized, withdrawn and intact rats using the elevated plus-maze (EPM), also, to re-challenge with a low dose of METH (0.5 mg/kg) in withdrawn groups.

**Results:** Results have shown that METH-sensitized rats exhibited an increase in the open arm time and entries 120 min after injection compared to the control group. We found a reduction in the time spent in open arms for the immediate effects of METH (30 min after injection) in METH-sensitized rats as compared to the control group. In withdrawn rats, METH/METH groups exhibited an increase in the open arm time and entries than METH/Sal and Sal/METH groups.

**Conclusion:** It was found that unlike delayed effects, an immediate effect of METH exhibited anxiogenic-like behaviors in METH-sensitized rats using the EPM. Also, results indicated that a low dose of METH is a potent stimulus for reinstatement of methamphetamine behavioral sensitization in a long withdrawn period.

Methamphetamine (METH) is one of the most harmful and addictive drugs, whose abuse is rapidly accelerating (1-3). Methamphetamine's fast brain uptake (9 min) is consistent with its highly reinforcing effects, its slow clearance (4), according to a plasma half-life of approximately 12 hours in humans (2) and 70 min in rats (5, 6) with its long-lasting behavioral effects and its widespread distribution (2) in cortical, subcortical and white matter areas of the brain with its neurotoxic effects compared with psych stimulants such as cocaine and amphetamine (2, 4). Therefore, METH can cause long-term changes in the brain structure and function and can also alter synaptic plasticity (2). In human studies, there are reports of anxiolytic effects (7), anxiogenic effects of METH during intoxication (8) and withdrawal (9), but the prolonged use of METH causes dependence and withdrawal syndromes such as anxiety and depression-like behaviors in humans, animals and the velvet monkeys (3,10-14). In animal studies, it has been shown that immediate (acute) METH injection (1mg/kg, 30 min prior to testing) decrease anxiety in the open field and the elevated plus maze (15). Also, a recent study has shown that administration of low-dose methamphetamine (3) or continuous subcutaneous administration with osmotic mini-pumps (15 or 76 mg/kg of METH) or under an escalating-dose injection regimen (0.2–2.0 mg/kg, 3 times daily for 4 days) (11) and low and high doses of METH alone (16) do not produce any behavioral changes such as anxiety. However, there are controversies over METH effects on anxiety in the literature. Thus, the effect of METH on anxiety is probably due to the full dose, duration of drug exposure and time- dependent effects (immediate and delayed effects) and different methodological approaches, particularly with respect to the selection of species (rat or mouse).

Lack of such knowledge prevents therapeutic intervention and reverses METH -induced neurotoxic damage. In this study, rats became sensitized to METH but did not develop dependence. Thus, one of the aims of this study was to examine whether methamphetamine -sensitized and withdrawn rats would show an increase in the expression of anxiogenic-like behaviors in EPM and METH- induced behavioral sensitization with 0.5 mg/kg of METH after a long period of withdrawal (15) as a neutral dose in the locomotor activity measurements and the EPM test. Also, this study examined the anxiety profile in rats 30 and 120 min after a single dose of METH injection (immediate and delayed effects).

## Material and Methods

*Animals and induction of methamphetamine- induced sensitization*

Adult male Wistar rats (200–250 g) were housed 4 per cage in a room with a 12 h light/dark cycle at 24 ± 2 °C and had ad libitum access to food and water. All experiments were performed between 10:00 and 12:00 during the light cycle. All of the experimental procedures were conducted in accordance with the National Institutes of Health’s Guide for the Care and Use of Laboratory Animals. All efforts were made to minimize the number of animals used and their suffering. Methamphetamine hydrochloride (Sigma–Aldrich, M 8750) was dissolved in 0.9% saline. The rats were chronically treated with subcutaneous injections of METH (2 mg/kg) once a day for 14 days as described previously (3, 17). Saline was similarly injected into control rats. All injections were made in a volume of 1 ml/kg. This dose of METH did not show any neurotoxicity but produced behavioral sensitization after repeated treatment in rats (18).

Anxiety Measurement in the Elevated plus Maze (EPM). The EPM test was carried out as described in our previous studies (14, 19).The EPM was a wooden plus-maze with two open arms (50×10 cm, with a ledge of 5 mm) and two closed arms (50×10×40 cm), which were linked by a common central platform (10×10 cm). The apparatus was placed at a height of 70 cm from the floor and illuminated by a 100 W desk lamp above the apparatus. The Rats were placed on the central platform facing an open arm and allowed to explore the apparatus for 5 min. The following variables were measured during each 5 min test: (1) time spent in open and closed arms as a percentage of the total time spent exploring both the open and closed arms; (2) the number of entries into the open and closed arms. The apparatus was cleaned with water after each trial. It should be noted that anxious rats of elevated open places entered the open arms less frequently and spent less time in the open arms compared to the closed arms when allowed to freely explore the EPM.

*Locomotor Activity Measurement*

To rule out the possibility that our behavioral effects in experiments 1 and 3 were attributable to either decrease or increase gross in movement, as well as to evaluate the behavioral sensitivity following withdrawal in experiment 3, we assessed locomotor activity rats immediately after the anxiety test. In experiments 1 and 3, spontaneous locomotor activity of each animal was measured using an automated activity monitor system (TSE infraMot, TSE, Bad Homburg, Germany) for 12 min (19). Only one animal was placed in each activity chamber per measurement time. 

*Experimental Protocol*

*Experiment 1*

**Figure 1:**
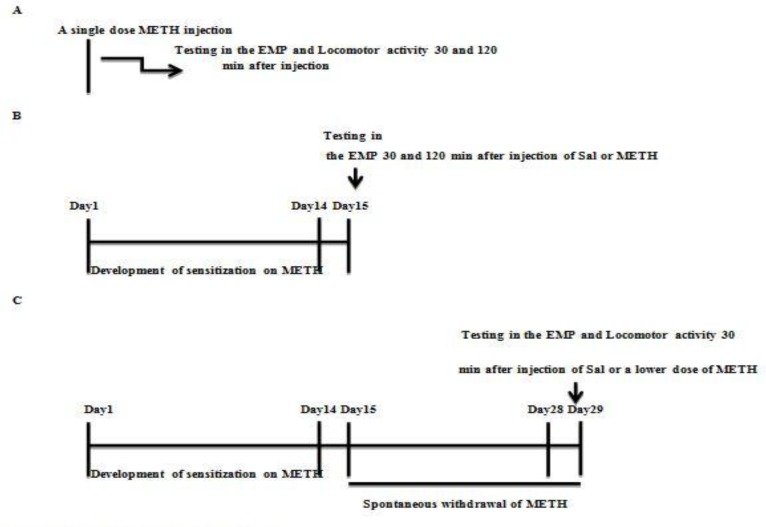
Timelines of experiments

This experiment evaluated the immediate and delayed effects of METH on the anxiety like- behavior in intact rats 30 and 120 min after a single dose METH injection (2 mg/kg, s.c.) considering the half-life of METH in rats (approximately 70 min(5, 6)). In this experiment, rats were randomly assigned into four groups (n = 7 rats per group): (Sal/30 min after injection), (METH/30 min after injection), (Sal/120 min after injection), (METH/120 min after injection). Immediately after the anxiety test, locomotor activity of all groups was tested during a 12- min period (Figure 1A. Timeline of the experiments)

*Experiment 2*

This experiment examined the immediate and delayed effects of METH on the anxiety like- behavior in METH-sensitized rats. In this experiment, 40 male rats were categorized into four groups and received saline or METH (2 mg/kg, s.c. for 14 days): Sal exposed/Sal/30 min after injection, (Sensitive to METH) METH exposed/METH/30 min after injection, Sal exposed/Sal/120 min after injection, METH exposed/METH/120 min after injection. All rats were tested on day 15 in the EPM, 30 and 120 min after a METH or saline injection (Figure 1B. Timeline of experiments).

*Experiment 3*

This experiment examined METH- induced behavioral sensitization with 0.5 mg/kg of METH after a 14-day period of withdrawal in METH-sensitized rats. A lower dose (0.5 mg/kg) (20) of the drug was used to maximize the differences in responsiveness to METH after withdrawal. Induction of methamphetamine- induced sensitization was done like experiment 2 for 14 days. On day 15, rats were placed in their home cages with no injection for 14 days (drug abstinence). On day 29, rats were randomly assigned into four groups: Sal/receiving saline (Sal/Sal), Sal/receiving METH (Sal/METH), METH-withdrawn rats receiving saline (METH/Sal), METH -withdrawn rats receiving METH (METH/METH). All animals were tested in the EPM 30 min after receiving METH or saline injections. Immediately after the anxiety test, locomotor activity of each animal was measured using an automated activity monitor system during a 12- min period (Figure 1C. Timeline of experiments).

*Statistical Analysis*

The data from anxiety tests were expressed as the mean standard error of the mean (S.E.M.). These data were analyzed using one-way analyses of variance (ANOVA). Post hoc analyses included Tukey’s test. Statistical differences were considered significant at P<0.05.

## Results

Experiment 1: Immediate effects (30 min after injection) of a single dose METH (2 mg/kg, s.c.) lead to increase in the severity of the anxiogenic-like behaviors in intact rats. 

Figure 2; Shows the results of a single dose METH injection in intact rats in the EPM test. One -way ANOVA revealed a significant difference in the number of open (F 3, 24= 80.95, P < 0.0001) closed arm (F 3, 24= 139.88, P < 0.0001) and total arms entries (F 3, 24= 85.34, P < 0.0001) (Figure 2A). 

Between-group comparisons indicated that the number of entries into the open arms of the intact rats 120 min after METH injection was significantly higher than saline and 30 min after METH injection group (P < 0.0001, both). Also, the number of entries into closed arms was significantly higher 30 min after METH injection than saline and 120 min after METH injection group (P < 0.0001, both). The number of total arm entries was significantly higher 30 and 120 min after METH injection compared to saline group (P < 0.0001, both).

Also, one -way ANOVA revealed a significant difference in the percentage of time spent in the open (F 3, 24= 174.56, P < 0.0001) and closed (F 3, 24= 143.53, P < 0.0001) arms (Figure 2B). Between-group comparisons indicated that the percentage of time spent in the open and closed arms 30 min after METH injection were significantly lower and higher than saline (P < 0.0001, P < 0.0001, respectively) and 120 min after METH injection group (P < 0.0001, both ).

Also, a single dose METH injection 30 and 120 min after METH injection in intact rats increased their locomotor activity compared to the saline group (P < 0.0001, both) (Figure 2C).

**Figure:**
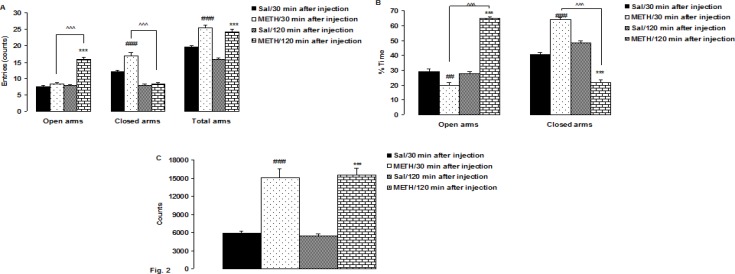


**Figure:**
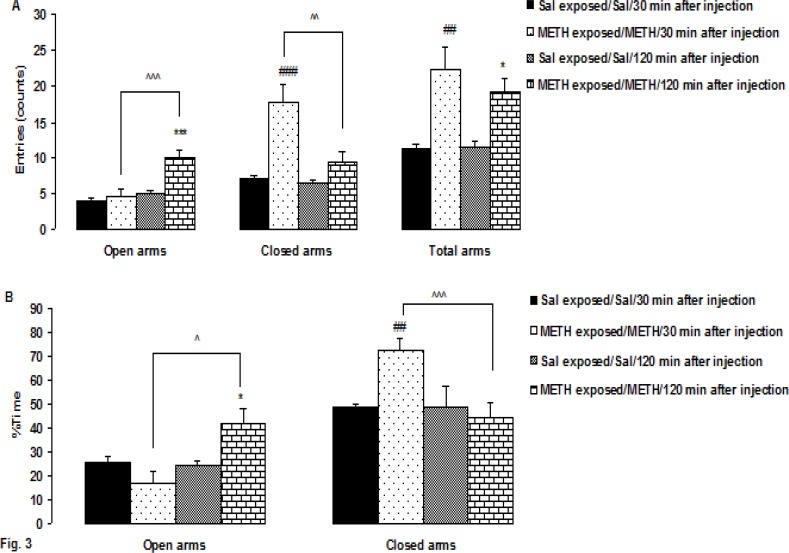


**Figure:**
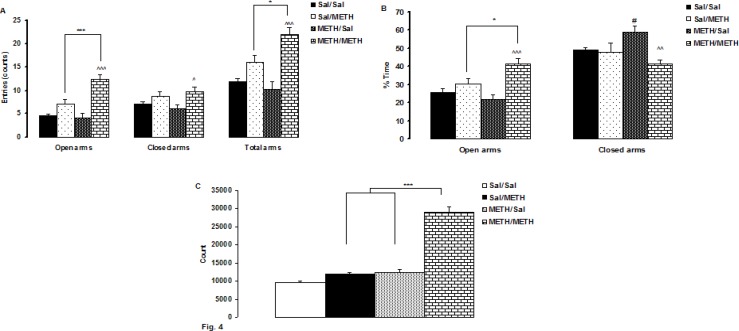


Figure 3. Shows the results of the EPM testing in METH-sensitized groups 30 and 120 min after injection. One -way ANOVAs revealed a significant difference in the number of open (F 3, 36= 11.79, P < 0.0001) and closed arm (F 3, 36= 12.64, P < 0.0001) and total arms entries (F 3, 36=9.91, P<0.0001) (Figure 3A). Between-group comparisons indicated that METH-sensitized rats (30 min after injection) had a significant increase in the number of closed arm entries than saline group (P < 0.0001). Also, METH-sensitized 120 min after injection had a significant increase in the number of entries into the open arms 120 min after injection than saline and 30 min after METH injection (P < 0.0001, P < 0.002, respectively). The number of total arm entries 30 and 120 min after METH injection in METH-sensitized rats were significantly higher than saline group 30 and 120 min after METH injection (P < 0.0001, P<0.021, respectively).

Figure 3B shows that METH-sensitized rats 30 and 120 min after injection had exhibited a significant difference in the percentage of the time spent in open (F3, 36 = 6.76, P < 0.001), closed arm (F 3, 36 = 11.23, P < 0.0001). 30 and 120 min after injection. Comparisons between the groups inThe METH-sensitized rats 30 min after injection spent significantly more time in the closed arms than saline group 30 min after injection (P < 0.001). Also, sensitized rats 120 min after METH injection spent significantly more time in the open arms 120 min after METH injection than saline and 30 min after METH injection (P < 0.017, P < 0.021, respectively). Sensitized rats 120 min after METH injection spent significantly lower time in the closed arms 120 min after METH injection than 30 min after METH injection group (P < 0.0001).

Results in the EPM in figure 4A shows a significant difference in the number of open (F 3, 36 = 23.82, P < 0.0001), and the total arms entries (F 3, 36 = 15.67, P < 0.0001) after a 14- day drug withdrawal in METH- sensitized rats followed by a low-dose METH injection. Between-group comparisons indicated that a low-dose METH injection in METH/METH rats after 14 days of withdrawal significantly increased the number of open (P < 0.0001, both) and the total arms entries (P < 0.0001, P < 0.012, respectively) than METH/Sal and Sal/METH rats, and decreased the number of closed arm than METH/Sal (P < 0.017) .

Figure 4B shows that the injection of a low dose of the drug in METH- sensitized rats after 14 days of drug withdrawal revealed a significant difference on in open (F 3, 36= 10.23, P < 0.0001) and closed arm (F 3, 36= 4.96, P < 0.006) times. Comparisons between group indicated that METH/METH rats spent significantly more time in the open arms than Sal/METH and METH/Sal (P<0.014, P < 0.0001, respectively);) , and also lower less time in the closed arms than METH/Sal (p< 0.003). Also, METH/Sal rats spent significantly more time in the closed arms than Sal/Sal (p< 0.007).

The results of the locomotor activity measurement showed are presented in figure 4C. One-way ANOVA for the locomotor activity showed a significant difference between the groups following saline or a low-dose METH (F 3, 36=63.71, P < 0.0001). Between-group comparisons showed that that locomotor activity of METH/METH group was significantly higher than METH/Sal and Sal/METH groups (P < 0.0001, both).

## Discussion

Our results of the EPM testing revealed that a single dose of METH injection in intact rats after 30 min of injection increased anxiety, and rats made significantly fewer open arm entries and spent less time in the open arms than the control group. This may be explained, in part, by activation of the sympathetic nervous system via alpha receptor stimulation, a putative METH -specific mechanism (9) and a decrease in the rate of dopamine and 5-HT uptake 30 min after exposure to the drug (21). This finding seems to contradict with a study that showed 1 mg/kg of METH s.c. 30 min prior to test decreased anxiety in the open field as well as in the EPM (15). This discrepancy may be due to dose-related effects of drugs. However, it seems that the effect of METH on anxiety is related to stressful factors since another study indicated that all acute doses of METH (0.5, 1 and 1.5 mg/kg) using open field 30 min after injection had an anxiogenic effect by reduced social interaction (22, 23). Overall, these studies showed that acute dose of METH 1 mg/kg increased social anxiety experienced in the face of foreign animal, while decreased anxiety after exposure to a new environment (15). Thus, one possible explanation for this inconsistency is the dose and time course of METH. 

Also, in this study, like others, the acute dose of METH increased total arms entries and locomotion (23). However, there are other studies showing that acute dose of METH 1 mg/kg decreased total arms entries (24), and decreased locomotor activity in the test of social interaction. On the other hand, in the test of social interaction, there was an increase of locomotion after single low-dose METH injection (22). Therefore, changes in locomotor activity induced by a single low-dose METH were related to the test environment. There was no significant difference in the locomotor activity between the two groups (30 and 120 min after METH injection), suggesting that reduced open arm activity in 30 min after injection or increased open arm activity in 120 min after injection were due to increased or decreased anxiety and not hyperactivity. 

Also, our results revealed that a single dose METH injection in intact rats after 120 min of injection decreased anxiety compared to the control animals. This may be explained by the fact that the half-life of METH is 70 min in rats (5, 6), showing recovery from the immediate effects of METH intoxication.

Experiment 2: Immediate effects of methamphetamine injection (30 min after injection) lead to increased anxiety-like behavior in sensitized rats. 

Our results have shown that immediate effects of METH injection in sensitized rats 30 min after injection compared with 120 min after injection increased anxiety-like behavior. This finding is consistent with the study showing that a long-term, escalating dose of METH procedure increased anxiety-related behavior in the velvet monkeys (13). Thus, the effect of METH on anxiety is probably related to the dose and duration of its administration. Also, the results of a study showed that a dose of METH 1 mg/kg decreased comforting behavior in prenatally treated groups by decreasing time spent by social interaction (15, 22). Therefore, another explanation may be that the rats developed tolerance to the METH until 120 min after injection, and this resulted in a reduction in drug efficacy (25). Therefore, anxiety was less at 120 min after injection (delayed effects). The other probable explanation is that aggressive behavior was more after 30 min of drug injection, while it clearly decreased 120 min after injection of the drug in sensitized rats and animals were quiet (Data is unpublished), and this may be due to the fact that the half-life of METH is 70 min in rats (5, 6). As in experiment 1, we did not observe any aggressive behavior after administration of a single dose of the drug. Our findings are consistent with a study in which the METH-induced fighting was significantly increased 15 min after METH but not 20 h post drug (26). There was no significant difference in the number of total arm entries between the two groups (30 and 120 min after METH injection), suggesting that reduced open arm activity at 30 min after injection or increased open arm activity at 120 min after injection were due to increased or decreased anxiety and not hyperactivity. It seems that dopamine D2 receptors play an important role in the expression of sensitized behaviors of METH (27).

Experiment 3: A low-dose methamphetamine (0.5 mg/kg) induces an anxiolytic reaction and hyper locomotion after 14 days drug withdrawal in METH- sensitized rats.

In our study, there were no differences in the locomotor activity between METH/Sal and Sal/METH after 14 days of withdrawal from METH. However, a low dose of METH produces an enduring hyper locomotion in METH-withdrawn rats. This phenomenon is termed behavioral sensitization. Behavioral sensitization has been proposed as a useful model for the intensification of drug craving, leading to a high rate of relapse in psych stimulant addiction (28, 29). In a previous study, 0.5 mg/kg of METH enhanced motor activity after a 7-8 day withdrawal (30), while in our study after 14 days of withdrawal from METH induced an increase of the locomotor activity, indicating the development of behavioral sensitization, and the duration of METH withdrawal which is generally considered more than 2 weeks.

Also, our results indicated that saline group receiving saline (Sal/Sal) and METH group receiving saline (METH/Sal) after 14 days drug withdrawal had no differences in the number of open and closed arm and total arms entries. In our study, motor activity was decreased after 14 days drug withdrawal in METH group receiving saline (METH/Sal). These findings suggest that the effects of 14 days of withdrawal from the drug has declined and this is consistent with a study showing changes in normal behavioral activities after METH exposure in utero which probably do not persist until adulthood (15). 

The administration of a low-dose methamphetamine (0.5 mg/kg) significantly increased open and total arms entries in the METH group receiving METH (METH/METH) compared to the METH group receiving saline (METH/Sal) and saline group receiving METH (Sal/METH). Also, it significantly decreased the closed arm time in the METH/METH group than METH/Sal group, probably by making exploration more rewarding through a mechanism unrelated to anxiety (31). Therefore, injection of a low-dose methamphetamine (0.5 mg/kg) is a potent stimulus for reinstatement of METH locomotors sensitization after 14 days drug withdrawal and possibly reinstatement of METH seeking in relapse. This finding is consistent with the results of a study that showed the rats exposed to METH in utero exhibited increased sensitivity to the effects of acute METH in adulthood, which increased the rewarding value of exploration in the open field (15). Behavioral hypersensitivity induced by low dose indicates that the duration of METH withdrawal is much longer. The duration of amphetamine withdrawal is generally considered more than 2 weeks than cocaine withdrawal, and this finding was approved in previous studies as well (9, 32). One possible explanation for this behavioral sensitization is that METH-induced increase in the dopamine of nucleus accumbens, the meso-accumbens, the ventral tegmental area and nigrostriatal dopamine system and chronic activation of dopamine D1 receptors mediate the effects of behavioral sensitization (15, 24, 29, 33). Also, behavioral sensitization and the facilitating role in the reinstatement of METH-seeking behaviors may be explained, in part, by increasing the corticotrophin releasing factor levels in the amygdala without the participation of corticosterone during withdrawal from METH (34). Also, our findings indicated that METH/Sal rats significantly increased anxiety- like behavior after 14 days withdrawal from the drug. This finding is consistent with our previous study showing that a 30-day withdrawal period increased anxiety-like behavior in METH-dependent rats (14). Also, one of limitation of our study was the lack of the neurobiological mechanisms and that should be considered in future studies.

## Conclusion

Our results revealed that immediate effects of METH exhibited anxiogenic-like behaviors while delayed effects of METH exhibited anxiolytic effect in METH-sensitized rats as assessed by the EPM. Also, results indicated that METH-sensitized rats showed anxiety-like behavior after 14 days withdrawal from the drug and a low dose of METH (0.5 mg/kg) induced an anxiolytic reaction and reinstatement of METH behavioral sensitization after a long period of abstinence in METH-withdrawn rats. Our study suggests that there are more psychological risks following the immediate injection of METH and also in METH-sensitized individuals which should be considered in the treatment. 
